# Gold Bead Implantation in Acupoints for Coxofemoral Arthrosis in Dogs: Method Description and Adverse Effects

**DOI:** 10.3390/ani2030426

**Published:** 2012-09-04

**Authors:** Gry T. Jæger, Øyvind Stigen, Morten Devor, Lars Moe

**Affiliations:** Department of Companion Animal Clinical Sciences, Norwegian School of Veterinary Science, P.O. Box 8146 Dep., 0033 Oslo, Norway; E-Mails: gry.jaeger@nvh.no (G.T.J.); oyvind.stigen@nvh.no (Ø.S.)

**Keywords:** acupuncture, side effects, canine, gold wire, degenerative joint disease, hip dysplasia, pain treatment

## Abstract

**Simple Summary:**

Traditional acupuncture uses needles inserted at certain acupuncture points. Gold bead implantation at acupuncture points is used in acupuncture intended to relieve pain in certain diseases. The method of gold implantation is not well described in the literature. We describe the technique of implanting 24-karat gold beads around the joints of dogs with degenerative joint disease due to chronic hip dysplasia. The method is safe and fairly easy to perform under anesthesia. It has few serious side effects, as long as bead-deposition within the joint is avoided. There is some aggravation of discomfort during the first two weeks after treatment as well as bleeding and synovial leakage during treatment.

**Abstract:**

Gold bead implantation has been used for years as an alternative method to improve function in chronic joint disease both in humans and dogs. The aims of the present study were to describe the technique of implanting 24-karat gold beads around the hip joints of dogs with chronic hip dysplasia, and to record any side effects or complications of such treatment. A prospective placebo-controlled double-blinded clinical trial was performed. Eighty dogs were randomly allocated to treatment or placebo, with 38 in the gold implantation group and 42 in the placebo group, and followed intensely for six months. The implantation technique was simple to perform, using fluoroscopy and with the dogs under inhalation anesthesia for about 30 minutes. Adverse effects, measured as pain or discomfort, were seen for a period of up to four weeks in 15 of the dogs in the gold implantation group, compared to six dogs in the placebo group. During implantation, a technical difficulty occurred as 82% of the dogs showed leakage of blood and/or synovia from the needles. The dogs in the gold implantation group were radiographed 18 months later. Of the 30 dogs that were radiographed at both inclusion and 24 months, 80% (24 dogs) showed a deterioration of the coxofemoral arthrosis, the other six had stable disease evaluated by radiography. Migration of gold beads was only observed in one dog.

## 1. Introduction

Gold bead implantation (GBI) is a treatment used in acupuncture to relieve pain in chronic joint disease. Traditional acupuncture uses needles inserted through the skin at certain acupuncture points and left in place for 20–30 minutes. The gold implants are inserted through large gauge needles at acupuncture points, and left in the body. Veterinarians have used gold bead implantations for many years [1,2]. Insufficiently documented reports from the USA [3] and Europe [4,5] claim to show extraordinarily good results for this procedure both in dogs and humans. The patients have shown less discomfort, improvement of movement, and even complete recovery from lameness. In addition, many anecdotal stories report favorable effects of GBI in both humans and animals with different chronic diseases, especially degenerative joint diseases (DJD). Two double-blinded and placebo-controlled clinical trials [6,7] failed to show any statistically significant difference between the GBI and the placebo groups for dogs with chronic hip dysplasia (HD). However, a more recent double-blind trial with 80 dogs was able to show a significant reduction in pain and gait abnormality in the GBI group [8]. 

We could not find any detailed descriptions of the GBI technique or any report on subsequent adverse effects in the scientific literature. The aim of the present article was to describe such a technique and to record any undesired effects caused by HD gold bead implantation in dogs suffering from chronic degenerative joint diseases. The clinical outcome of the treatment has been published elsewhere [8,9].

## 2. Materials and Methods

### 2.1. Animals

The dogs (n = 80) had a radiographic diagnosis of either unilateral or bilateral HD and no other causes of pain or hind limb lameness. Hip dysplasia was graded as mild, moderate or severe according to the guidelines of the Scientific Commission of the Nordic Kennel Union (NKU) and the Federation Cynologique Internationale (FCI). The dogs were randomly assigned to two groups. Thirty-eight dogs were treated with GBI and forty-two dogs were used as controls. In the treated group fifteen dogs had severe bilateral HD, three had severe unilateral HD. Eighteen dogs had mild or moderate bilateral HD, and two had unilateral HD. In the control group 19 animals had severe bilateral HD, one had severe unilateral HD, and 22 dogs had mild or moderate bilateral HD. 

Follow-up radiographs of the hip region were taken immediately after the implantation, at the six months evaluation when the code was broken, and at 24 months. Two radiologists independently evaluated the radiographs for any changes in the hip joint arthrosis and any possible migration of the gold beads, by comparing radiographs taken at different times. The numbers and positions of the gold beads were recorded at implantation and at six and 24 months post-implantation. The identification of the dogs and the dates of the radiography were hidden from the evaluators. Of the 38 dogs in the GBI group, 36 dogs were evaluated radiologically. Six of the dogs were only radiographed at the six-month follow-up evaluation.

The National Ethical Committee for Animal Care approved the study and the owners gave their written consent.

### 2.2. Preparation and Anesthesia before Implantation

**Figure 1:**
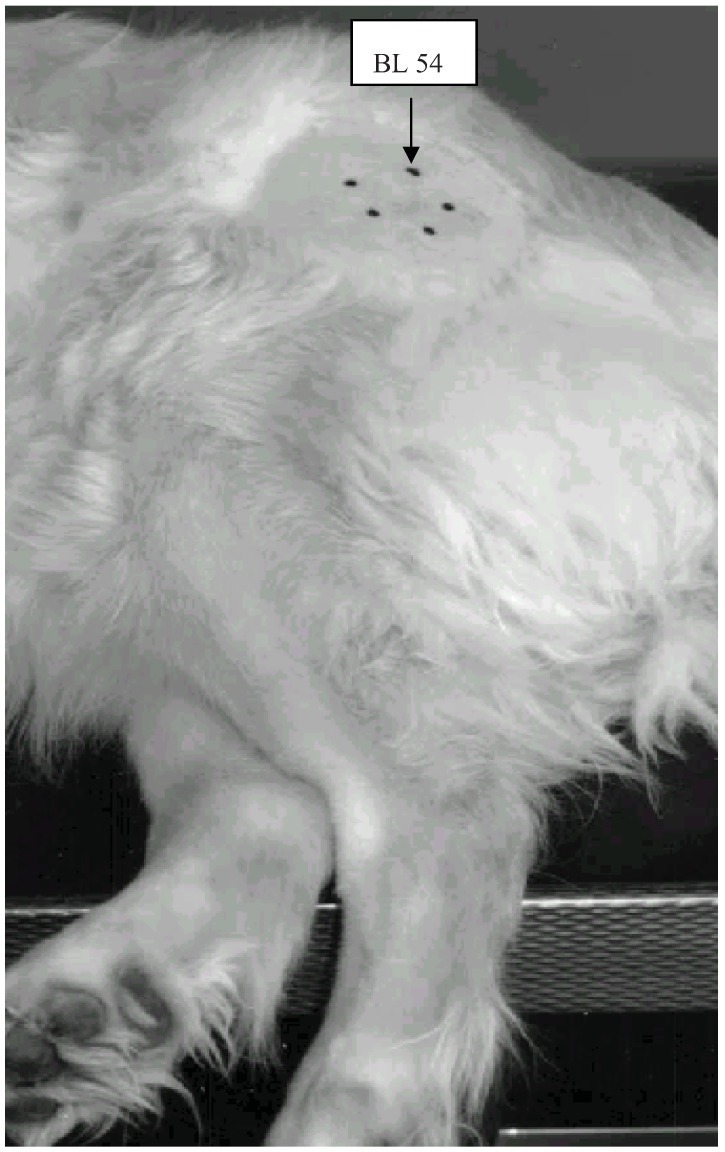
The acupuncture points GB 29, GB 30 and BL 54 together with two trigger-points marked with Indian ink in the hip area.

An area of approximately 10 cm × 10 cm was clipped over each affected hip joint and aseptically prepared with chlorhexidine soap (Hibiscrub® solution 40 mg/mL) (Figure 1). A veterinarian certified in veterinary acupuncture used an ohmmeter or a “point finder” [10] to find the implantation points. Five points were identified as the acupuncture points GB 29, BL 54 and GB 30 in positions 9, 12 and 3 o’clock with the *trochanter major* as the center, and two trigger points (painful points) in positions 5 and 7 o’clock. The acupuncture and trigger points were called Points 3, 5, 7, 9 and 12 after their clockwise positions. The area was disinfected with chlorhexidine and ethanol 5mg/ml (Galderma® color ethanol solution). 

After premedication with xylazine (Narcoxyl® 20 mg/mL, ”Veterinaria”) 1.0 mg/kg i.v., anesthesia was induced with propofol (Rapinovet® 10 mg/mL “Schering-Plough”) 4.0 mg/kg i.v., and the dogs were intubated. Anesthesia was maintained with propofol in the first 20 dogs and with 2% isoflurane (Forene® “Abbott”) in oxygen (150 mL/kg) in the remaining dogs. The mean duration time of anesthesia was 32 minutes if both hips were treated (range 15–60 minutes).

### 2.3. Implantation of Gold Beads and Fluid from Needles

**Figure 2:**
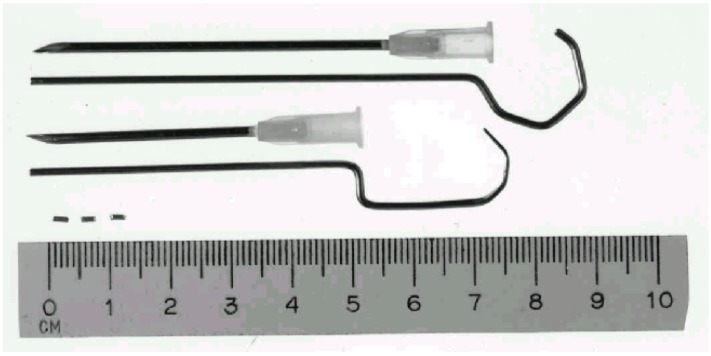
Three gold beads and two different sizes of 14 G needles together with their adapted stylet.

Twenty-four karat gold wire (1 mm in diameter), obtained from an Oslo jeweler, was cut into 2 mm beads (Figure 2). Each bead weighed 35–40 mg. The gold beads and a stylet were packed and autoclaved. Two sterile gold beads were implanted in each of the five defined acupuncture or trigger points (Figure 1).

**Figure 3:**
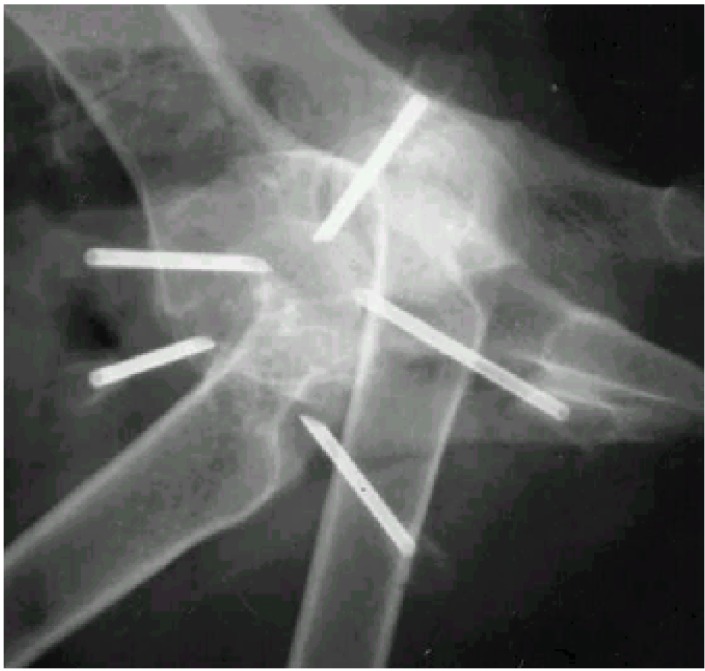
Fluoroscopic evaluation during placement of the needles.

**Figure 4:**
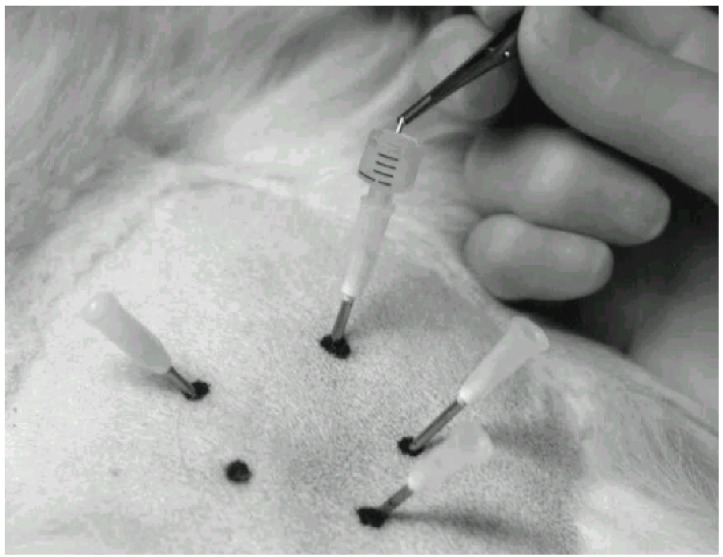
One gold bead is dropped into a needle using forceps and a funnel.

Stainless steel needles (Hypojet® 60 mm × 2.0 mm, 14 G × 2½") (Figure 2) were inserted at an oblique angle at each point and directed towards the joint capsule of the femoral head. The position of the needle tip was adjusted under fluoroscopy (Figure 3). Acupuncture point GB 30 (Point 3) is close to the sciatic nerve. Damage to the nerves was avoided. Any synovial fluid or blood observed from the needle was recorded, and a new needle was reinserted a few mm in another direction. When all the five needles were in place, two gold beads were dropped into each needle through a funnel made from a syringe (Figure 4). A stylet of the same length as the needle was used to push the gold beads just beyond the needle tip and the needle was withdrawn. The skin wounds were covered with liquid plaster (Wound Plast® “Karex”). Both hips were treated in dogs with bilateral HD.

### 2.4. Study Design

The study was designed as a prospective placebo-controlled double-blind clinical trial where the clinician and dog-owners were blinded to the treatment given. Only family-owned dogs showing pain and difficulty with ambulation due to HD were included. Thorough clinical examinations were performed before, two weeks, three months and six months after entering the trial. The dogs (47 females and 33 males) were allocated by block-randomization to the “GBI group” and the “placebo group”. The dogs represented 28 different breeds and had a mean weight of 34.8 kg. Age varied from one to eight years (mean 4.4 years). The same procedures were used in all the dogs in both groups. In the control group, the procedure ended when the needles had penetrated the skin. No GBI was performed, and no metal was deposited. 

### 2.5. Pain after Gold Bead Implantation

Pain aggravation after GBI was scored subjectively by owners according to the following definitions: no aggravation of pain signs (constant lameness, no change in discomfort, no change in avoiding jumping or climbing stairs, *etc.*); mild aggravation of pain lasting up to seven days; moderate aggravation of pain lasting up to 14 days; severe aggravation of pain lasting up to four weeks. 

### 2.6. Statistical Analysis

All results are expressed either in numbers of observations or percents. If fluid leaked from the needle, it was classified as either blood or synovia depending on the color and viscosity. The presence of fluid is expressed either per dog or per needle. The dispersions are indicated with 95% confidence interval (CI), calculated using the theory of single binominal sequences [11]. All tests were performed two-tailed with a significance level of 5%. Comparisons of groups were performed with contingency table analysis [11].

## 3. Results

Blood and/or synovia from the implantation needles were recorded in 81.6% of the dogs (Table 1). Blood was observed significantly more frequently than synovia; 73.7% (CI 56.9–86.6) *versus* 36.8% (CI 21.8–54.0). Twenty dogs with mild or moderate HD had gold implantations through 190 needles, while 165 needles were used in 18 dogs with severe HD (Table 2). Synovia leakage was significantly more frequent in severe HD compared to mild or moderate HD (*P *< 0.05), but there was no significant difference for blood. Synovia leakage was seen most frequently at Point 12, and was significantly more common than at Point 3 (*P *= 0.05). Blood was found most frequently at Point 5, which was significantly higher than at Point 9 (*P *< 0.01). When comparing the number of dogs with any type of leakage with respect to mild or moderate HD *versus* severe HD, no significant difference was found between the groups (Table 1).

**Table 1:** Number of dogs with blood and/or synovia leakage from the implantation needles during insertion of gold beads around the hips among 38 dogs with hip dysplasia.

Type of fluid	Mild or moderate hip dysplasia	Severe hip dysplasia	Total
Blood	11	6	17
Synovia	3	0	3
Blood and synovia	3	8	11
No fluid	3	4	7
Total number of dogs	20	18	38

**Table 2:** The percentage of blood and synovia leakage from the needles during implantation of gold beads at five acupuncture points (Point 3–12, see Figure 1) around the hips of 38 dogs with hip dysplasia (HD). The percentage is calculated as the number of observations divided by the total number of needles multiplied by 100 (N = 355).

Fluid leakage	Hip dysplasia (number of needles)	Percentage of implantation needles with blood and/or synovia (95% confidence interval)					
		Point 3	Point 5	Point 7	Point 9	Point 12	Total
*Blood*	Mild or moderate HD	3.7	4.7	2.1	0.5	1.1	12.1
	(n = 190)	(1.0–6.4)	(1.7–7.8)	(0.1–4.2)	(0–2.1)	(0–2.9)	(7.5–16.7)
	Severe HD	3.6	5.5	3.0	1.2	3.0	16.4
	(n = 165)	(0.8–6.5)	(2.0–8.9)	(0.4–5.7)	(0–3.3)	(0.4–5.7)	(10.7–22.0)
*Synovia*	Mild or moderate HD	0.5	0	1.1	0	2.6	4.2
	(n = 190)	(0–2.1)		(0–2.9)		(0.4–4.9)	(1.4–7.1)
	Severe HD	1.2	3.0	1.2	3.6	3.0	12.1
	(n = 165)	(0–3.3)	(0.4–5.7)	(0–3.3)	(0.8–6.5)	(0.4–5.7)	(7.1–17.1)

**Table 3:** Pain aggravation in dogs with hip dysplasia after treatment in gold implantation group *versus* placebo group of dogs with blood and/or synovia leakage from the implantation needles during insertion of gold beads around the hips of 38 dogs with hip dysplasia.

	Pain aggravation				
Treatment group	None	Mild Lasting up to 7 days	Moderate Lasting up to 14 days	Severe Lasting up to four weeks	Total
Gold implantation	23	9	4	2	38
Placebo	36	5	1	0	42
Total number of dogs	59	14	5	2	80

Twenty-one of the 80 owners (26.3%) reported pain aggravation recorded as discomfort, stiffness or lameness for up to four weeks after the implantation (Table 3). Pain aggravation was reported significantly more frequently (*P *= 0.036) in the GBI group (39.5% (CI 24.0–56.6)) compared to the placebo group (14.3% (CI 5.4–28.5)). Of the 21 dogs showing post-implantation pain, four from the GBI group and one from the placebo group were given analgesics such as NSAIDs for up to eight days.

No signs of infection at the implantation sites were reported by the owners or were observed by the veterinarian at the follow-up visits.

The evaluation of the radiographs showed that two gold beads of a total of 710 (355 × 2) beads had obviously migrated in one dog when comparing the position of the gold beads on the initial radiograph with the follow-up radiographs. The gold beads had migrated three and six centimeters, respectively, in a median plane around the same hip. This dog showed no post implantation pain.

Of the 30 dogs that received implants, 24 (80%) showed increased secondary new bone formation on the 24-month follow-up radiographs, compared with the radiographs taken at the time of implantation. No reduction in periarticular bone formation was observed. No difference in increased secondary osteoarthritis between mild or moderate HD *versus* severe HD was observed.

## 4. Discussion

Although gold implantation has been used for many years to treat a range of conditions, both in dogs and humans [4], thorough scientific descriptions of the methodology are scarce. A detailed description of any adverse effects of the treatment is also lacking. In cases of chronic DJD in dogs, the reported level of efficacy of gold bead treatment in relieving pain and restoring gait has varied considerably [2,3,6,7,12].

We modified a method originally published by Durkes (1992) [2,3], and subsequently employed by Klitsgaard (1996) [5], and others.

Since 24-karat gold was the metal most often used, and since Klitsgaard [5] and others used it in Denmark, 24-karat gold was chosen in the present study. Durkes claimed that a magnetic metal plated with gold gave better results [3], but we were unable to find published accounts that confirm this. The use of other metals could elicit allergic reactions, which we wanted to avoid. There may be better results with other metals or alloys, but until the clinical effect of 24-karat gold is well characterized, we think it is reasonable to first get solid scientific data using gold.

The clinical significance of the ultimate location of the gold beads and points of entry of the needles are not previously discussed in the literature. These questions were not addressed in a systematic way in our study, but they are highly relevant. The choice to use five needles, inserted in three traditional canine acupuncture points and two trigger points, using two gold beads per location [2], were arbitrary decisions. We also decided that the gold beads should be located close to the hip joint capsule, similar to locations described by others [2,3]. The “correct” position of the tip of the needles was facilitated by the use of fluoroscopy (Figure 3). If fluoroscopy is not available, it may be helpful to take radiographs and ensure that the tip of the needle is adjacent to the hip joint capsule before the gold beads are deposited.

The number of gold beads per needle varies between reports. The common procedure seems to employ three beads per needle [2,3]. One study used only one and the reason was not discussed [7]. We decided to use two gold beads per needle, but have no documented rationale to believe that one or three gold beads could not work as well.

We presumed that it was not important where the needles entered the skin, but still we chose to use acupuncture points. An electrical point-finder was used to identify the points. Durkes [1] emphasized that it was very important that acupuncture points be used, although Klitsgaard [5], who employed both acupuncture and non-acupuncture points with equal efficacy, questioned this opinion. If there is a significant effect of the treatment, we believe that this is more likely due to the ultimate positioning of the gold beads and not the method of delivery through the skin.

There were few long-term side effects of the GBI treatment such as pain, infection and migration of the gold beads, but technical difficulties may occur during positioning of the needles. One is leakage of blood or synovia from the insertion needles. To our knowledge no data exist on how frequently this may occur, but it is mentioned as an occasional finding [2,3]. In the present study, leakage was commonly observed and only seven of 38 dogs did not show any leakage from any needle (Table 1). However, when expressed in terms of number of needles, 4%–16% of the needles showed fluid leakage (Table 2). Blood was recorded more often than synovia, which may be explained by the large diameter of the needles.

Synovia leakage was seen significantly more often in dogs with severe HD than with moderate or mild HD. The reason is probably that severe HD cases have more distended joint capsules containing more fluid that are more easily punctured than the moderate HD cases. Careful attention during GBI in dogs with severe HD is recommended. If the needle penetrates the joint capsule, the risk of deposition of the beads into the joint is high. It may lead to severe pain, and should certainly be avoided [2,3].

Pain was reported significantly more often by owners after GBI compared to the placebo group. Increased discomfort and lameness were also reported during the first days after treatment in the GBI group. Others have also found mildly increased lameness in dogs treated with GBI, and those dogs had significantly decreased peak vertical force and vertical impulse on a force plate at least one month following GBI [6]. A human study confirmed that patients experienced some local pain for a few days post-implantation and occasional hematomas were seen [4]. A canine study described that signs of dysfunction and pain abated within a week of GBI in most dogs [2]. The study by Klitsgaard (1996) [2] was not performed as a prospective clinical trial and side effects were not properly evaluated with evaluation forms within 14 days after implantation, and the dogs were not re-examined by a veterinarian at follow-up visits. In our study, however, almost 40% of the dogs treated with GBI showed some aggravation of discomfort and lameness during the seven days after GBI. The pain aggravation subsided during the following week. Several explanations might be given for this discrepancy. 

The most probable explanation for the reported pain is that the gold beads induce a mildly painful tissue response. We do not know the nature of that response, but one might speculate that inflammation or disturbance of nociceptors may be involved. Inflammatory reactions around the gold beads in dogs were clearly seen in one histopathology report [13]. There are many nerve endings in the joint capsule, and if either the capsule was penetrated or some of the beads lay in contact with it, pain might result. Since an increased aggravation of pain was also seen in dogs in the placebo group, it is possible that stretching of the hips during positioning for radiography in anesthesia may be responsible. It is also possible that the GBI dogs were manipulated more than the controls during the positioning of the needles. An extra mechanical distress to the degenerated joint could be an additional explanation for why the GBI group had a longer lasting and more severe aggravation than the placebo group. None of the gold beads in our study were positioned within the joint capsule, but other authors have experienced such misplacement with severe pain and lameness as the result [2,3].

There are very few reports in the literature of migration of gold beads. Two dogs were radiographed in one study [2] and six dogs in another uncontrolled case report [12], and no migration was observed with certainty. In our study a migration of two gold beads was seen unilaterally in one dog. The dog, a St. Bernard, was only one year old at the time of implantation. Thus, the dog was still growing which could be the reason why migration over several centimeters was observed. A migration may likewise occur if the gold bead is deposited into an interfacial space. It may also be difficult to interpret the exact position of the beads on a two-dimensional radiograph. In mature dogs, however, migration of gold beads over several centimeters seems to be rare.

Among some veterinarians with long experience in gold implantation there is a comprehension that the GBI treatment will slow down or even reverse the DJD of HD and new bone deposition [3]. Durkes (1992) claimed that after having treated more than 250 dogs, decalcification around the hip joints could be discerned in some dogs six to twelve months after implantation [3]. There is, however, a lack of exact observations supporting this conclusion. The present study shows a completely different result. Approximately 80% of the dogs showed increased new bone formation after 18–24 months and no dog had reduced bony radiographic pathology as evaluated by two independent radiologists. Our findings are in agreement with a case report where two of five dogs showed increased hip joint degenerative disease, and three had the same degree of DJD, after six months to three years [12].

Severe infection at the implantation site is the most common complication due to improper aseptic technique, according to one report [3]. No data in that publication supported the conclusion. In our study, and before the randomization result was revealed to the owners, they were encouraged to be aware of any signs of infection. Although antibiotics were not used prophylactically, we did not experience any signs of infection in any of the dogs after GBI. We may therefore conclude that a normal surgical preparation procedure will effectively prevent any noticeable infection and there is no need for antibiotics in normal dogs. 

## 5. Conclusions

The method of GBI is safe and easy to perform. With the exception of some aggravation of discomfort during the first two weeks post-implantation, the method has few serious side effects, as long as bead-deposition within the joint is avoided.
